# Edible coatings of chitosan and fucoidan with crucian carp protein hydrolysate to extend shelf life of beluga sturgeon fillets

**DOI:** 10.1016/j.heliyon.2025.e42296

**Published:** 2025-01-27

**Authors:** Khadijeh Jafari Malekkolaei, Sara Jafarian, Seyed Rohollah Javadian, Seyedeh Khadijeh Mahdavi, Somayeh Bahram

**Affiliations:** a Food Science and Technology Department, Nour Branch, Islamic Azad Univercity, Nour, Iran; b Department of Fisheries, Qaemshahr Branch, Islamic Azad University, Qaemshahr, Iran; c Islamic Azad University, Nour Branch, Department of Natural Resource, Nour, Iran

**Keywords:** *Carassius carassius*, Enzymatic hydrolysis, Fish quality, *Huso huso*, *Sargassum latifolium*, Nanoliposome

## Abstract

This study investigates the problem of preserving the quality and shelf life of beluga sturgeon (*Huso huso*) fillets under refrigeration. The aim of the research is to evaluate the effectiveness of composite edible coatings made from chitosan and fucoidan, in combination with crucian carp (*Carassius carassius*) protein hydrolysate (CPH). Significant methodology includes the preparation of hydrolyzed proteins using the enzyme Alcalase for 90 min, followed by encapsulation within nanoliposomes. Fucoidan was extracted from the brown alga *Sargassum latifolium*, and its antioxidant and antimicrobial properties were assessed. Six types of edible coatings were developed, including chitosan and chitosan-fucoidan with CPH and nano CPH (NCPH) at concentrations of 0 %, 0.5 %, and 1 %. The chemical, microbiological, and sensory characteristics of the coated and uncoated fish fillets were evaluated over a 16-day storage period. Results indicated a particle size of 102.98 nm, a zeta potential of 12.29 mV, and an encapsulation efficiency of 75.84 %. The extraction yield of fucoidan was 5.89 %, with total carbohydrate content of 59.2 %, protein content of 9.21 %, sulfate content of 21.78 %, and uronic acid content of 8.85 %. Fucoidan exhibited significant antioxidant and antimicrobial activity. The analysis of chemical and microbiological factors in the fish fillets revealed significantly lower values in the coated fillets compared to the control (P < 0.05). The use of CPH significantly reduced the rate of oxidative spoilage, and increasing CPH concentration resulted in a slower rise in microbial load (P < 0.05). The best outcomes were observed in NCPH. Ultimately, the combination of chitosan-fucoidan and 1 % NCPH maintained acceptable conditions for most microbiological, chemical, and sensory indices by the end of the storage period. These findings suggest that these edible coatings could serve as an effective method for enhancing the quality and shelf life of fish fillets in the food industry.

## 1 Introduction

One of the primary challenges faced by food producers today is maintaining product quality during storage. Biodegradable films and coatings, typically made from biopolymers, can significantly extend the shelf life of food products [[1], [2], [3]]. These coatings serve as protective layers, preventing direct exposure to environmental factors such as air and moisture [[4], [5], [6]]. Additionally, the use of active edible films as a novel packaging method allows for the controlled release of bioactive compounds, thereby enhancing the shelf life of food products [[7], [8], [9]]. Marine algae account for 58 % of the global production of aquatic plants and are widely cultivated along the coasts of Asia. One prominent species is the brown alga Sargassum latifolium, which contains valuable compounds such as polysaccharides. These polysaccharides have garnered attention due to their unique properties and are utilized across various industries, including food, pharmaceuticals, and cosmetics [10,11]. Fucoidan, a significant polysaccharide found in brown algae, is recognized as a bioactive compound [12]. It exhibits outstanding properties, including antioxidant, antiviral, anticancer, and anti-inflammatory activities. Specifically, fucoidan's ability to combat free radicals and reduce inflammation highlights its potential for use in functional foods and dietary supplements [13,14]. Additionally, the biological properties of fucoidan, such as its ability to form antimicrobial films and its biodegradability, make it suitable for applications in the food and health industries [13,14].

Since each individual component of a film may have certain weaknesses, combining different materials can improve the properties of edible films and coatings. Chitosan, a natural polysaccharide derived from chitin found in crustacean shells, is widely used in the food and pharmaceutical industries. This polymer is an effective barrier to gas transmission, particularly oxygen, due to its semi-permeable nature, and plays a significant role in enhancing the preservation of food products [[3], [15]]. Chitosan not only extends shelf life and enhances the quality of food products under anaerobic conditions but also possesses antimicrobial and antioxidant properties that can be further improved by incorporating natural materials like hydrolyzed proteins [16,17]. Moreover, due to the presence of positive functional groups in its structure, chitosan is biodegradable and hygienic, making it an ideal option for various applications [[3], [15]]). Chitosan coatings can delay enzymatic browning, reduce water loss, preserve natural flavor, and improve the texture and color stability of food products [18]. Therefore, integrating fucoidan with chitosan can significantly enhance the properties of edible films and coatings, leading to the development of more effective food packaging solutions [14,18,19].

Crucian carp (*Carassius carassius*), a freshwater fish in the carp family, holds limited commercial value due to its small size and undesirable taste and odor. However, it is considered an important protein source in some countries. This fish is often viewed as a nuisance in warm-water fish farms because it competes for food with carp. Its low price and high production levels make it an attractive candidate for processing into value-added products [20].

The increasing interest in natural antioxidants has spurred extensive research into the antioxidant capabilities of peptides derived from hydrolyzed proteins, such as those from soy, wheat, and milk [21,22]. This process, which can be considered beneficial for producing high-value-added products, employs both chemical and biological methods. Enzymatic hydrolysis not only allows for precise control over production but also aids in recovering bioactive peptides with low molecular weights, which are easily absorbed by the body and play significant roles at the cellular level [[23], [24], [25]]. These peptides are particularly noteworthy for their antioxidant properties, including their ability to scavenge free radicals and inhibit fat oxidation [22,26,27].

As active compounds, peptides may lose their beneficial properties upon exposure to oxygen or food components. Therefore, protective methods are essential to preserve their high antioxidant activity. One effective approach is the encapsulation (or nanoencapsulation) of peptides prior to application in food systems, significantly enhancing their stability and effectiveness [28,29]. Nanoliposomes have emerged as a novel method for delivering drugs and bioactive compounds, capable of simultaneously carrying both hydrophilic and hydrophobic components within a single vesicle. This enhances the stability, functional properties, cellular absorption, and controlled release of compounds. Liposomes can be easily produced from edible ingredients and can encapsulate both polar and non-polar amino acids found in hydrolyzed proteins [[29], [30]].

Fish and fishery products can meet a significant proportion of the world's food needs by 2050 [31]. I Beluga sturgeon (*Huso huso*) is the largest fish species in the Caspian Sea and belongs to the sturgeon family. This species has garnered significant attention due to its high nutritional value and unique characteristics [21]. The aim of this study is to develop composite edible films using chitosan, fucoidan extracted from brown algae, and hydrolyzed proteins and nanoproteins from crucian carp to protect beluga sturgeon fillets from oxidation and microbial spoilage during cold storage. A key objective of this research is to examine the chemical, microbiological, and sensory changes in sturgeon fillets and compare them with control samples. This study seeks to enhance understanding of the quality characteristics and shelf life of sturgeon fillets under cold storage conditions and to investigate the role of biodegradable edible coatings in preserving quality and extending shelf life.

## 2 Materials and methods

### 2.1 Preparation of raw materials

Beluga sturgeon (*Huso huso*) was sourced from a fish market in Babolsar, Mazandaran, Iran. After purchase, the fish was cut into small pieces and transported on ice to the Caspian Sea Ecology Research Institute. A total of 72 fish fillets, each weighing between 80 and 100 g, were prepared for treatment selection. The brown alga *Sargassum latifolium*, native to the coastal regions of the Persian Gulf in Bushehr Province, was collected, cleaned, and washed several times with freshwater. It was then dried in an oven at 38 °C, ground into powder using a grinder, and stored in plastic bags at −20 °C until use. Crucian carp (*Carassius carassius*), averaging 100 g, were sourced from a market in Sari, Mazandaran, Iran. The enzyme Alcalase, an endoprotease with a specific activity of 4.2 Anson units per g and a density of 18.1 g/mL, was obtained from Bacillus licheniformis and purchased from Novozymes, Denmark, and stored at 4 °C until use. Ethanol, buffer solutions, Tris–HCl, hydrochloric acid, and DPPH (2,2-diphenyl-1-picrylhydrazyl) radicals were acquired from Merck, Germany, with all chemicals being of analytical grade.

### 2.2 Preparation of hydrolyzed protein from crucian carp

The fish was deboned, skinned, and internal organs were removed, followed by washing with water. The flesh was ground three times using a meat grinder and stored in zippered plastic bags in a freezer at −20 °C until further use. The frozen ground fish was thawed overnight in a refrigerator. The sample was weighed and subjected to enzyme inactivation by placing it in a water bath at 85 °C for 20 min. The heat-treated sample was mixed with a Tris-HCl buffer solution (1:2, w:v) to create a uniform suspension with a pH suitable for Alcalase activity (pH = 8.5). Hydrolysis was conducted at 50 °C with an enzyme concentration of 1 % in 100 mL flasks in a shaking incubator set at 200 rpm for 90 min. To ensure complete enzyme deactivation, the reaction mixture was heated at 85 °C for 10 min and then cooled in an ice bath. The suspension was centrifuged at 8000 g at 10 °C for 20 min to collect the supernatant, which was then stored in a freezer and subsequently freeze-dried to obtain a powder [32].

### 2.3 Protein quantification

The protein content was determined following AOAC [33] guidelines based on the kjeldahl (K1100, Hanon, China) method. To assess the degree of hydrolysis, the hydrolyzed protein suspension was mixed with 0.44 M trichloroacetic acid in a 1:1 vol ratio and incubated at 4 °C for 15 min. The mixture was centrifuged at 10,000 rpm for 10 min, and the protein concentration in the supernatant containing trichloroacetic acid was calculated using the following formula [34]:

Equation 1 Degreeofhydrolysis(%)=(Proteininhydrolysatesuspension/ProteininsupernatantcontainingTCA)×100

To determine the amino acid profile, each sample was hydrolyzed with 6 N hydrochloric acid at 110 °C for 24 h. After centrifugation at 1200 g at room temperature for 3 min, derivatization with o-phthalaldehyde (OPA) was performed, and amino acid analysis was conducted using fluorometric methods The amount of total amino acids was measured using a smart line HPLC device (Knauer, Germany) and using a C18 column with a fluorescent detector (RF-530) [22].

### 2.4 Preparation of nanoliposomes containing bioactive peptides

The hydrolyzed protein was subjected to ultrafiltration using a Darmstadt Merck KGaA Millipore EMD system (Germany) to obtain peptides with a molecular weight below 3 kDa. For the preparation of nanoliposomes, the hydrolyzed protein (10 mg/mL) was mixed with phospholipid/cholesterol in hydrated distilled water and glycerol, heated to 60–80 °C. The pH of the mixture was adjusted to 3.7 using 2 M sodium hydroxide and maintained at 60 °C with continuous shaking for 1 h. The crude liposomes were homogenized under high pressure and prepared in 1 % (w/v) acetic acid at room temperature (25 °C) while being mixed at 200 rpm for 1 h. The liposomal solution was sonicated at 25 °C using an ultrasonic device (200 UPs) for further processing. Nanoliposomes were stored under nitrogen gas at room temperature for subsequent experimental steps [28].

The particle size and distribution of the liposomes were measured after diluting the sample tenfold with buffer using dynamic light scattering (DLS) with a Zetasizer (Malvern Nano ZS, UK) [35]. The surface zeta potential of the liposomes was measured using the Zetasizer, with the liposomes diluted tenfold with phosphate buffer (74, 50 mM) and analyzed at a scattering angle of 173° and a helium-cadmium laser wavelength of 633 nm at room temperature [35].

The encapsulation efficiency (EE%) was determined based on the method of Muangrat et al. (2012) with modifications. For this, 100 mg of the encapsulated powder was combined with 1 mL of 0.1 M potassium phosphate buffer (pH 8) and vortexed for 15 min. The surface protein content (SPC) in the encapsulated powder was measured using the Bradford method. The encapsulation efficiency (EE%) was calculated using the following formula:

Equation 2 $$\text{Encapsulation}\;\text{efficiency}\;\left(\text{EE}\%\right)=\frac{TPC-SPC}{TPC}\times 100$$

### 2.5 Extraction and characterization of fucoidan

20 g of dried seaweed powder were subjected to two extraction rounds with 400 mL of pure ethanol for 3 h at room temperature, with constant stirring to remove unwanted compounds. The biomass was then washed with distilled water and dried in a vacuum oven at 45 °C until a constant weight was reached. To extract fucoidan, the biomass was treated with a solvent-to-seaweed ratio of 20 mL/g at 45 °C for 160 min using a heated stirrer. The resulting mixture was cooled on ice and neutralized to pH 7 using 2 M sodium hydroxide. The precipitate was separated by vacuum filtration, and the supernatant was treated with double the volume of pure ethanol at 4 °C overnight to precipitate fucoidan. The precipitate was collected by centrifugation at 7000*g* for 15 min at 4 °C and purified by washing twice with 70 % ethanol, then dried in a vacuum oven at 45 °C [36].

The extraction yield was calculated using the following formula [37]:

Yield(percentage)=Dryweightofalgae(g)/Dryweightoffucoidan(g)×100

The sulfate content of fucoidan was determined after hydrolysis in 0.5 M hydrochloric acid at 105 °C for 5 h, following the method described by Lee et al. [38], with barium chloride (BaCl) and potassium sulfate (K2SO4) as standards. The protein content was measured using the Lowry method with a protein assay kit [39]. Total carbohydrate content was measured based on the method mentioned by Gorji et al. [39], using an absorbance measurement at 630 nm. Uronic acid content was determined following the procedure of Filisetti-Cozzi and Carpita [40], using glucuronic acid as a standard.

### 2.6 Antibacterial activity assessment

The antibacterial activity of fucoidan was assessed using the disk diffusion method against *Staphylococcus aureus* (ATCC 29737) and *Escherichia coli* (ATCC 10536). A 24-h bacterial culture was prepared in nutrient broth, and a microbial suspension equivalent to the 0.5 McFarland standard was made. Then, 100 μL of this suspension was added to Mueller-Hinton agar. Uniform bacterial growth was spread on the agar surface using a sterile glass rod. A blank disk impregnated with 30 μL of the extracted seaweed compound was placed on the agar surface. Plates were incubated at 37 °C for 24 h, and antibacterial activity was measured by assessing the zone of inhibition using a Vernier caliper (accuracy: 0.2 mm) in triplicate [41].

### 2.7 Radical scavenging activity measurement

The radical scavenging activity was measured based on the method of Gharekhan Taghar Tapeh et al. (2020). A 5.23 mg solution of DPPH was prepared in 100 mL of absolute methanol. 0.1 mL of this solution was mixed with 9.3 mL of DPPH solution in sterilized tubes, vortexed for 1 min, and kept in the dark at 25 °C for 30 min. The absorbance of the samples was measured at 517 nm by a spectrophotometer (Thermo Fisher Scientific, Wilmington, DE, USA).

Equation 3 DPPHradicalscavenging%=(Absorbancecontrol/Absorbancesample−Absorbancecontrol−1)×100

### 2.8 Preparation of chitosan-fucoidan composite coating

To prepare the composite coating, chitosan was used at a concentration of 2 %, alongside fucoidan at 1 %. These components were combined with varying concentrations of hydrolyzed protein and nanoprotein (0.5 % and 1 %). The mixture was dissolved in 200 mL of distilled water and heated to 70 °C for 45 min while stirring continuously using a stirrer [17]. This process ensured a homogeneous composite coating suitable for application on fish fillets.

### 2.9 Sample preparation

Fish fillets weighing between 80 and 100 g were immersed in the composite coating (2 % chitosan and 1 % fucoidan) with varying concentrations of protein and nanoprotein hydrolyzed (0.5 % and 1 %) for 1 min. The samples were then removed from the solution and allowed to drain for 30 s to remove excess water. Next, they were immersed in a 2 % calcium chloride solution for 30 s to promote cross-linking in the coating [17]. Subsequently, the samples were placed in sterile zip-lock bags, while control samples were similarly stored. All samples were then kept at 4 °C in a refrigerator. Chemical and microbiological tests were conducted on days 0, 4, 8, 12, and 16 of storage.

### 2.10 Oxidative Index measurements

#### 2.10.1 Peroxide value determination

The peroxide value was determined according to the procedure outlined by Javadian et al. [42]. A sample of extracted fish oil was weighed and placed in a 250 mL Erlenmeyer flask. Approximately 25 mL of a chloroform-acetic acid solution (in a 2:3 ratio) was added. Next, 0.5 mL of saturated potassium iodide solution, 30 mL of distilled water, and 0.5 mL of 1 % starch solution were incorporated. The liberated iodine was titrated with a 1 % sodium thiosulfate solution until the endpoint was reached.

#### 2.10.2 Thiobarbituric Acid (TBA) measurement

The TBA content was assessed using a colorimetric technique. A 200 mg portion of the fish sample was placed in a 25 mL flask and diluted with 1-butanol to the mark. Five mL of this solution was transferred to dry test tubes and combined with 5 mL of TBA reagent, prepared by dissolving 200 mg of TBA in 100 mL of 1-butanol and filtering. The test tubes were incubated in a water bath at 95 °C for 2 h. After cooling to room temperature, the absorbance (As) was measured at 530 nm against a blank (Ab) containing distilled water [31]. The TBA value, expressed in mg of malondialdehyde per kg of fish, was calculated using the following equation:

Equation 4 TBA=(As−Ab)×50/200

#### 2.10.3 Total Volatile Basic Nitrogen (TVB-N) measurement

The TVB-N content was determined according to the method described by AOAC [33]. A 10 g sample of fish fillet was combined with 2 g of magnesium oxide and 300 mL of distilled water in a Kjeldahl flask. Glass beads and a small quantity of n-octane (as an antifoam agent) were added. The flask was then attached to a distillation apparatus and heated. In a separate 250 mL Erlenmeyer flask, 25 mL of a 2 % boric acid solution (2 g of boric acid in 100 mL of distilled water) was prepared, along with a few drops of methyl red indicator (0.1 g in 100 mL ethanol). Methyl red changes color from red in acidic conditions to yellow in basic conditions. Distillation continued for 30 min or until approximately 125 mL of distillate was collected in the Erlenmeyer flask. The boric acid solution turned yellow, indicating alkalization due to volatile basic nitrogen. The resulting solution was titrated with 0.1 N sulfuric acid until it reverted to red. The TVB-N value, reported in mg per 100 g of fish fillet, was calculated using the following equation:

Equation 5 TVB−N=Volumeofsulfuricacidused×1.4×100/Weightofsample

#### 2.10.4 Microbiological testing

To evaluate the antimicrobial effects of the edible coating on the microbial load of fish samples during refrigerated storage, total viable count (TVC) and psychrotrophic bacterial count (PTC) were measured. For this purpose, 25 g of the sample were homogenized in a sterile environment with 225 mL of 0.1 % peptone water for 60 s using a blender at room temperature. Serial dilutions were prepared up to 106,106, using 0.1 % peptone water in a 1:10 ratio. From each dilution, 0.1 mL was spread onto agar media for bacterial culture. Total viable count was determined using PCA agar, with samples incubated at 30 °C for 48 h. For the psychrotrophic bacterial count, incubation was done at 7 °C for 10 days [[19], [43]]. To increase accuracy, three replicates of each sample were analyzed, and four suitable dilutions were evaluated per day for each replicate. The counts were converted to the logarithm of colony-forming units (CFU).

#### 2.10.5 Sensory evaluation

Sensory evaluation was conducted using quantitative descriptive analysis (QDA) with a panel of 20 evaluators, consisting of staff and students from the Faculty of Food Science and Technology at Islamic Azad University, Nour Branch, Iran. The panel was diverse in terms of age and educational background, adding credibility to the evaluation process. Prior to starting the analysis, training sessions were held for the evaluators to familiarize them with the vocabulary and scales used in the sensory assessment. The sensory characteristics evaluated included odor, color, taste, and overall acceptance, using a 5-point hedonic scale for scoring. To reduce flavor transfer between samples and enhance evaluation accuracy, water was provided for the evaluators to cleanse their palates before tasting each sample [44].

### 2.11 Statistical analysis

All tests were performed in triplicate. Statistical analysis of the data was conducted using a completely randomized design, and mean comparisons were made using Duncan's multiple range test at a 95 % confidence level. The results were analyzed using SPSS software version 18 (SPSS Inc., Chicago, USA), and Excel 2010 software was used for figures [31].

## 3 Results and discussion

### 3.1 Initial protein content analysis in various treatments

The initial protein content in crucian carp and its hydrolyzed protein powder was reported as 17.01 ± 0.95 % and ranged from 83.23 % to 52.55 %, respectively (Table 1). These findings indicate that hydrolyzed carp protein contains significantly higher protein levels compared to raw fish, a result consistent with previous studies [27,29,32]. The increase in protein content can be attributed to moisture removal and the separation of non-protein components during hydrolysis and centrifugation. This process transforms proteins into more absorbable forms, enhancing their applications in the food and nutrition industries. For instance, Mehregan Nikoo et al. [20] reported an initial protein content of 16.43 % in crucian carp, increasing to 79.13 % after hydrolysis, underscoring the effectiveness of this method in enhancing the nutritional value of carp protein.

**Table 1:** Degree of hydrolysis and protein content of crucian carp protein hydrolysates.

Hydrolysis time (min)	Degree of hydrolysis (%)	Protein content (%)
30	13.99 ± 0.78^c^	52.55 ± 0.99^c^
60	19.95 ± 0.88^b^	71.39 ± 1.16^b^
90	28.32 ± 1.11^a^	83.23 ± 2.29^a^

a Values are presented as means ± standard error (SE) with n = 3.

b Values within the same column that have different lowercase letters are significantly different at P < 0.05.

### 3.2 Degree of hydrolysis analysis

Controlling the degree of hydrolysis is crucial as it influences the properties of hydrolyzed proteins, including free amino acids, solubility, and antioxidant properties. Results (Table 1) show that hydrolysis time significantly impacts the degree of hydrolysis, with the highest degree observed at 90 min and the lowest at 30 min. Notably, hydrolysis was also higher at 60 min. During the initial stages, the accessibility of protein chemical bonds allows water to break them more efficiently. The increase in amino acid concentration and reduction in peptide molecular weight during these early stages enhance solubility, with enzymatic activity peaking at this time. Similar results have been reported regarding hydrolyzed fish proteins using commercial enzymes, indicating that the degree of hydrolysis increases significantly, especially during the initial phases [[20], [22], [26],^27^,^29^,^32^,^45^].

### 3.3 Amino acid composition

Fish protein hydrolysates are recognized as valuable sources of absorbable peptides and amino acids with multiple health benefits, including antioxidant, anti-inflammatory, anticancer, and antibacterial effects [29,46]. All essential amino acids recommended by WHO/FAO (1990) for adult daily consumption were present within acceptable limits. Glutamic acid, a key amino acid in fish, was the most abundant non-essential amino acid at 9.78 % (Table 2). Lysine, an important essential amino acid, was found at 13.05 %, marking it as the most prevalent essential amino acid and highlighting the potential of this fish as a quality protein source [46]. The total amount of hydrophobic amino acids in the hydrolyzed proteins reached 41.25 %, indicating significant nutritional value. Hydrophobic amino acids such as leucine, isoleucine, phenylalanine, and valine are essential for maintaining protein structure and contribute to favorable physical and chemical properties. These amino acids also act as precursors for synthesizing bioactive compounds, enhancing antioxidant activity, and regulating blood sugar and pressure [47]. According to Luo et al. [46], the optimal amino acid composition of crucian carp protein shows great potential for applications in the food industry and nutritional health enhancement.

**Table 2:** The amino acid composition crucian carp protein hydrolysates (g/100 g).

Amino acid		[48]
Histidine^a^	4.77	
Isoleucine^a^	5.88	2.8
Leucine^a^	11.21	6.6
Lysine^a^	13.05	5.8
Methionine^a^	3.24	
Phenyl alanine^a^	6.44	6.3
Tyrosine	4.24	1.1
Threonine^a^	5.28	3.4
Arginine	8.58	
Valine^a^	4.55	3.5
Aspartic acid	7.98	
Glycine	3.87	
Proline	3.48	
Serine	3.58	
Alanine	4.21	
Glutamic acid	9.78	
Total amino acid	98.14	
HAA^b^	41.25	

a Essential amino acids.

b Total hydrophobic amino acids (alanine, valine, isoleucine, leucine, tyrosine, phenylalanine, proline, methionine and cysteine).

### 3.4 Evaluation of nanoliposome tests

article size plays a crucial role in determining the stability, encapsulation efficiency, and release dynamics of bioactive compounds. Research has shown that variations in particle size significantly affect these properties [28]. In this study, the average size of peptide-loaded nanoliposomes was 102.98 ± 2.47 nm. Rashidi et al. [28] reported a similar size of 93.64 nm for nanoliposomes containing clover sprout peptides, highlighting the consistency of our findings. Minor differences may arise from variations in sonication time or the components present in the dispersion [35].

### 3.5 Zeta potential analysis

Zeta potential is critical for nanoparticle stability; values below +30 mV or above −30 mV indicate high stability [49]. In this study, the zeta potential was measured at 12.29 ± 0.89 mV, suggesting a suitable level of stability for the nanoparticles. This stability is vital for applications like nanoliposomes, as it prevents aggregation and sedimentation, maintaining their physical and chemical properties. A high zeta potential can positively influence the biological activity and absorption of bioactive compounds, underscoring the importance of optimizing this parameter in nanoparticle design [50].

### 3.6 Encapsulation efficiency

The encapsulation efficiency of nanoliposomes is influenced by their bilayer structure, which consists of hydrophilic and hydrophobic sections. Hydrophilic compounds are enclosed in the aqueous environment within the liposome, while hydrophobic compounds are embedded between phospholipid layers. This structure enhances encapsulation efficiency and stability against degradation and leakage of encapsulated compounds, particularly significant in food and pharmaceutical applications [50]. In this study, the encapsulation efficiency was found to be 75.3 ± 4.28 %, demonstrating effective performance comparable to Rashidi et al. [28], who reported an encapsulation efficiency of 68.20 % for nanoliposomes loaded with bioactive peptides derived from clover sprouts.

### 3.7 Evaluation of fucoidan tests

The extraction tests for fucoidan from the brown alga Sargassum latifolium yielded the following results: extraction yield was 5.89 ± 0.35 %, total carbohydrates were 59.31 ± 2.18 %, protein content was 9.21 ± 0.58 %, sulfate content was 21.78 ± 2.18 %, and uronic acid content was 8.85 ± 0.18 %. In comparison, Gorgij et al. [39] reported an extraction yield of 7.23 % for fucoidan from brown algae, with total carbohydrates at 36.51 %, protein content at 5.64 %, sulfate content at 11.57 %, and uronic acid at 9.22 %. Lee et al. [38] found extraction yields and contents of total carbohydrates, sulfate, uronic acid, and protein in fucoidan from Ecklonia cava to be 1.8 %, 51.8 %, 20.1 %, 11.3 %, and 8.7 %, respectively. The composition of fucoidans can vary significantly across different algal species due to extraction methods and experimental conditions [39,51].

### 3.8 DPPH radical scavenging activity

The DPPH radical scavenging activity of the extracted fucoidan was measured at 63.85 ± 2.74 %, indicating a strong ability to donate hydrogen atoms. Similar results were reported by Sahragard et al. [52], with DPPH scavenging activities for fucoidan extracted from Sargassum ilicifolium ranging between 63.27 % and 81.28 %, demonstrating a direct correlation with fucoidan concentration. Palanisamy et al. [53] noted 61.2 % scavenging activity for fucoidan from *Sargassum polycystum*. Gorgij et al. [39] found DPPH scavenging rates in fucoidan from *Sargassum tenerrimum* to vary between 35.94 % and 63.94 % at concentrations from 0.5 to 2 mg/mL.

### 3.9 Antimicrobial activity of fucoidan

The antimicrobial properties of fucoidan stem from various compounds, including amino acids, terpenoids, flavonoids, and phenolic compounds. In this study, fucoidan from *Sargassum latifolium* demonstrated antibacterial activity against *E. coli* (11.34 ± 1.24 mm) and *S. aureus* (13.75 ± 0.75 mm). The structure of fucoidan chains varies by algal species, affecting its functional properties against bacteria. For instance, G-negative bacteria possess a double glycoprotein layer that hinders the penetration of antibacterial agents, while compounds with lower molecular weight can more easily inhibit bacterial division in G-positive bacteria [54]. Fucoidan's structure may facilitate its ability to traverse bacterial walls or form gels on membranes, impeding nutrient exchange and inhibiting bacterial growth. With increasing bacterial resistance to conventional antibiotics, fucoidan presents a promising avenue for antimicrobial drug development [41,55]. These findings corroborate those of Gharekhan Taghar Tapeh et al. [41], who reported that fucoidan from *S. boveanum* effectively inhibited *E. coli* and *S. aureus.*

### 3.10 Peroxide Value (PV) during storage period

**Fig. 1:**
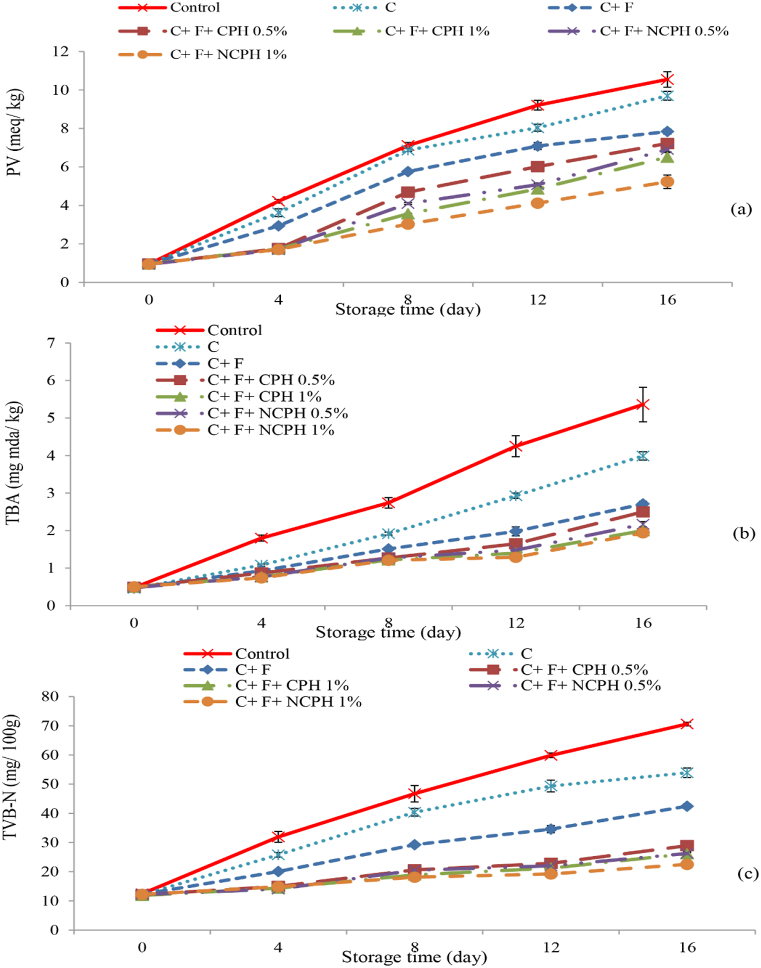
Peroxide value (PV), Thiobarbituric acid (TBA), and total volatile basic nitrogen (TVB-N) of beluga sturgeon fillets coated with chitosan (C), fucoidan (F), crucian carp protein hydrolysate (CPH), and nano CPH (NCPH) during storage.

Monitoring the peroxide value (PV) in fish samples is an effective method for assessing lipid oxidation and, consequently, the freshness and quality of the product [56]. In this study (Fig. 1a), PV increased across all treatments over the storage period, with the most significant changes observed in the control group (P < 0.05). This increase may be attributed to enzymatic reactions and microbial metabolic activity leading to lipid oxidation. The use of a chitosan coating significantly reduced the rate of PV increase compared to the control, highlighting chitosan's antioxidant properties and its ability to delay lipid oxidation in fish [17]. Chitosan forms hydrogen bonds and creates a protective layer around food, reducing oxidative damage and preserving seafood quality [15,57].

The addition of fucoidan to the chitosan coating further improved PV, likely due to fucoidan's natural antioxidant properties, which help react with free radicals and prevent lipid oxidation. Compounds in fucoidan, such as phenols and tannins, contribute to the reduction of hydroperoxide formation [14]. The best results for PV were noted in the chitosan + fucoidan +1 % nano-protein treatment, attributed to the antioxidant properties of hydrolyzed proteins. These proteins contain small peptides that can scavenge free radicals and prevent lipid oxidation, while also forming a protective layer to hinder oxygen penetration [42,58] (Tajik et al., 2023). The maximum allowable PV for fish intended for human consumption is 5 meq/kg of fat [59], and the chitosan + fucoidan + 1 % nano-protein treatment remained within acceptable limits throughout the storage period.

### 3.11 Changes in Thiobarbituric Acid (TBA) Value during storage

The TBA value is an indicator of secondary oxidation products such as malondialdehyde (MDA), which are formed during lipid oxidation reaction [60]. The initial TBA value gradually increased over the storage period for all treatments. During storage, the TBA (Fig. 1b) in the control was significantly higher than in other samples (P < 0.05), likely due to dehydration of the fish fillet and oxidation of unsaturated fatty acids [61]. The rise in TBA in the control group indicates lipid oxidation and degradation of unsaturated fatty acids, leading to decreased product quality. Chitosan and fucoidan coatings effectively reduced oxidation, maintaining seafood quality. Both compounds provide protective effects due to their antioxidant properties, and their combination has a synergistic effect in reducing lipid oxidation. Khorami et al. [62] also reported that adding fucoidan to a chitosan-alginate bilayer coating enhanced its antioxidant properties, positively affecting TBA changes in rainbow trout during storage. The lowest TBA values were observed in the chitosan + fucoidan +1 % nano-protein treatment, demonstrating the effective antioxidant properties of hydrolyzed proteins, which can trap metal ions and lower malondialdehyde (MDA) levels [63]. A TBA value of 2 mg MDA/g of meat is considered the consumer limit beyond which fish spoilage becomes detectable [64]. The chitosan + fucoidan + protein and nano-protein treatments remained within the acceptable range throughout the storage period.

### 3.12 Evaluation of total volatile base nitrogen (TVB-N) levels

Total Volatile Basic Nitrogen (TVB-N) concentration is a crucial indicator of meat freshness and quality, providing insights into flavor, color, and overall appearance [65]. As shown in Fig. 1c, TVB-N levels increased throughout the storage period for all treatments, with the control group exhibiting a significantly greater rise (P < 0.05). This increase can be linked to the growth of spoilage bacteria and the activity of endogenous enzymes, leading to undesirable byproducts such as ammonia [66]. The addition of fucoidan to the chitosan coating effectively reduced TVB-N increase, attributed to fucoidan's antimicrobial properties which inhibit bacterial growth and reduce enzymatic activity [41,62]. The lowest TVB-N values were seen in the chitosan + fucoidan +1 % nano-protein treatment. Hydrolyzed proteins, with their small peptides, can react with free radicals and bacteria, further preventing the formation of undesirable compounds and reducing TVB-N levels. Mighan et al. [63] reported similar findings, noting that hydrolyzed protein from watermelon seeds inhibited the increase in TVB-N levels in fish burgers.

The maximum allowable TVB-N value for fish fillets is set at 30 mg nitrogen/100 g [67]. On day 16 of storage, all treatments containing protein and nano-protein remained within the acceptable range.

### 3.13 Total viable count (TVC) and psychrotrophic bacterial count (PTC) during storage

**Fig. 2:**
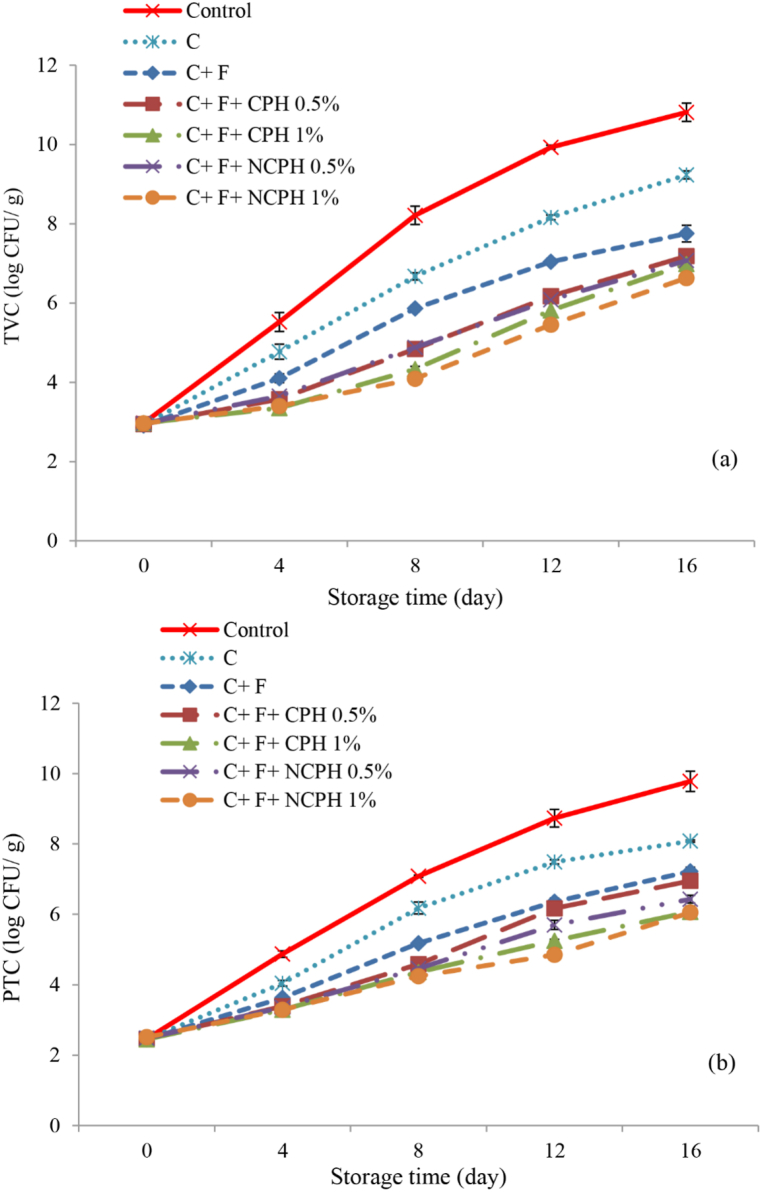
Total viable count (TVC) and Psychrotrophic bacterial count (PTC) of Beluga Sturgeon Fillets Coated with Chitosan (C), Fucoidan (F), Crucian Carp Protein Hydrolysate (CPH), and Nano CPH (NCPH) During Storage.

In this study, the total viable count (TVC) (Fig. 2a) and psychrotrophic count (PTC) (Fig. 2b) in fish fillet samples showed significant changes over the storage period (P < 0.05). The results indicated a notable difference in bacterial growth between coated and uncoated fillet samples. Specifically, fish fillets without a coating exhibited a more pronounced increase in bacterial counts, suggesting a protective effect of the coatings (P < 0.05). The application of a chitosan coating helped reduce bacterial load, highlighting its antimicrobial properties. Chitosan is a natural and biocompatible polymer known for its antimicrobial effects, which arise from its capacity to inhibit the growth of microorganisms and the activity of various enzymes. This antimicrobial effect is primarily due to the presence of positively charged amino groups and cationic components in chitosan's molecular structure. These features facilitate interactions with bacterial cell membranes, resulting in metabolic disturbances that can lead to membrane disruption and the release of intracellular substances [[68], [69], [70]].

Additionally, incorporating fucoidan into the chitosan coating has been shown to effectively reduce bacterial counts, including both total and psychrotrophic bacteria, in fish fillets. Fucoidan can interact with components of bacterial cell walls and cytoplasmic membranes due to its glycoprotein receptor structure. This interaction disrupts membrane permeability, leading to leakage of proteins and DNA damage in bacteria [62,71]. Khorami et al. [62] also reported that adding fucoidan to a chitosan-alginate dual-layer coating enhanced the antimicrobial properties of the coating, improving its effectiveness in controlling bacterial changes in rainbow trout during storage. The lowest counts of TVC and PTC during the storage period were observed in the treatment containing chitosan, fucoidan, and 1 % nano-protein (P < 0.05).

The antimicrobial activity of hydrolyzed proteins is attributed to small peptides and their active functional groups. The mechanism of action of these peptides primarily involves electrostatic interactions with microbial cell membranes. Antimicrobial peptides can increase membrane permeability, facilitating their entry and ultimately disrupting membrane functions, leading to microorganism death [72]. Additionally, nano-liposome encapsulation of hydrolyzed proteins can further enhance their antimicrobial effects. This technique increases the contact area and access of the proteins to bacterial cells. Nano-liposomes act as efficient carriers, enabling targeted delivery of proteins to microorganisms, thereby improving the quality and safety of food products and enhancing antimicrobial effects [29,73].

According to the International Commission on Microbiological Specifications for Foods [74], the maximum allowable TVC and PTC for fish is 7 log CFU/g. On day 16 of storage, all treatments containing hydrolyzed proteins and 1 % nano-protein remained within the acceptable range.

### 3.14 Sensory evaluation during storage

**Fig. 3:**
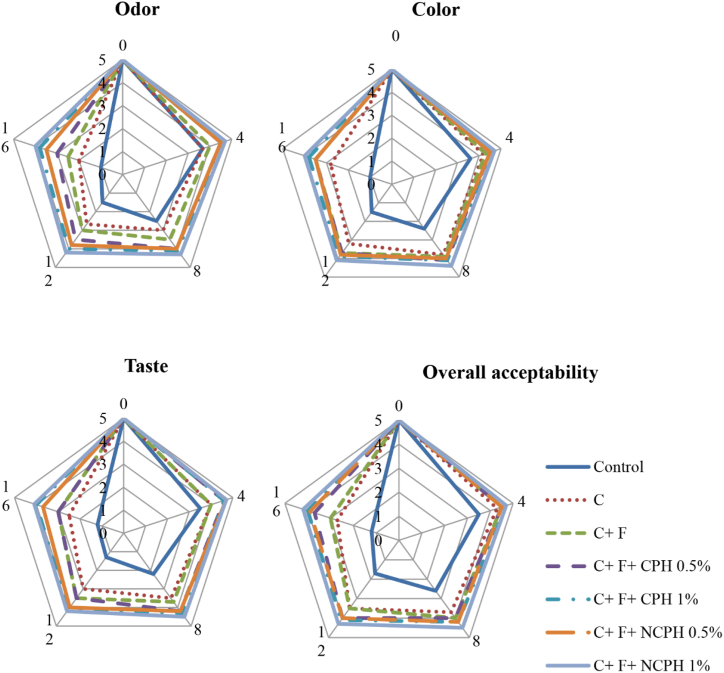
Sensory score of Beluga Sturgeon Fillets Coated with Chitosan (C), Fucoidan (F), Crucian Carp Protein Hydrolysate (CPH), and Nano CPH (NCPH) During Storage.

Sensory evaluation of fish is an essential method for assessing product quality and freshness, involving the examination of sensory attributes such as odor, taste, texture, and overall acceptance. This evaluation helps consumers make informed purchasing decisions, as products deemed unacceptable by evaluators are likely unsuitable for general consumption. Additionally, sensory evaluation (Fig. 3) can demonstrate the positive effects of additives and compounds like chitosan, fucoidan, and hydrolyzed proteins in improving the sensory characteristics of fish, contributing to the preservation of product quality and consumer appeal.

Throughout the storage period, there was a significant decline in sensory attributes across all treatments (P < 0.05). In the control group, an unpleasant odor was detectable after just 4 days of refrigeration, rendering the samples unsuitable for consumption at that time. These sensory changes aligned with the chemical and microbiological findings, suggesting that the decline in sensory quality was closely linked to oxidative processes and microbial spoilage. Notably, by day 16, the highest sensory ratings were recorded for the treatment that included chitosan, fucoidan, and 1 % nano-protein. The enhancement of sensory qualities in this treatment can be attributed to the synergistic effects of these three preservatives, which collectively exhibit strong antioxidant and antimicrobial properties. This combination effectively hinders lipid oxidation and microbial growth, thereby improving the taste, texture, color, and overall acceptability of the fish fillets [62,75,76].

Similar observations were made by Khorami et al. [62] regarding chitosan-fucoidan coatings on rainbow trout fillets and by Mighan et al. [63] concerning hydrolyzed watermelon seed protein in fish burgers. Both studies indicated that sensory evaluation results aligned with other chemical and microbiological parameters, showing that the use of these preservatives significantly improved the sensory qualities of the fish.

## 4 Conclusion

This study demonstrated that composite edible coatings containing chitosan and fucoidan, along with hydrolyzed nano-proteins from crucian carp, effectively enhance the shelf life and quality of fish fillets. At higher concentrations, these compounds significantly improved the chemical and microbiological properties of the fillets. Microbial and chemical analyses indicated that these coatings slowed oxidation and reduced microbial load during the 16-day storage period. Notably, the combination of chitosan, fucoidan, and 1 % nano-protein maintained favorable quality and sensory attributes by the end of the storage period. These findings underscore the high potential of these edible coatings in preserving the quality and safety of seafood products. As such, it is recommended that food manufacturers consider integrating these coatings into their preservation strategies. Future research should explore the scalability of this method in commercial applications and investigate the long-term effects of these coatings under various storage conditions. Additionally, studies on consumer acceptance and sensory evaluation could further support the implementation of these innovative solutions in the food industry, particularly in an era increasingly focused on natural and environmentally friendly ingredients. This research could pave the way for the development of sustainable and healthy food preservation methods.

## CRediT authorship contribution statement

**Khadijeh Jafari Malekkolaei:** Writing – original draft. **Sara Jafarian:** Writing – review & editing, Supervision. **Seyed Rohollah Javadian:** Formal analysis. **Seyedeh Khadijeh Mahdavi:** Resources. **Somayeh Bahram:** Software, Methodology, Investigation.

## Data availability statement

Data available on request from the authors.

## Ethics statement

This study involved sensory evaluations conducted in accordance with established ethical guidelines. Written informed consent was obtained from all participants prior to their involvement in the study.

## Declaration of competing interest

The authors declare that they have no known competing financial interests or personal relationships that could have appeared to influence the work reported in this paper.
